# Comparison of the Performance of 24 Severe Acute Respiratory Syndrome Coronavirus 2 Antibody Assays in the Diagnosis of Coronavirus Disease 2019 Patients

**DOI:** 10.3389/fmicb.2022.876227

**Published:** 2022-08-08

**Authors:** Shiji Wu, Hongyan Hou, Huijun Li, Ting Wang, Wei Wei, Minxia Zhang, Botao Yin, Min Huang, Ziyong Sun, Feng Wang

**Affiliations:** Department of Laboratory Medicine, Tongji Medical College, Tongji Hospital, Huazhong University of Science and Technology, Wuhan, China

**Keywords:** COVID-19, SARS-CoV-2, serological tests, lateral flow immunoassay, enzyme-linked immunosorbent assay, chemiluminescent immunoassay

## Abstract

**Background:**

The accurate detection of severe acute respiratory syndrome coronavirus 2 (SARS-CoV-2) is the key to control Coronavirus Disease-2019 (COVID-19). The performance of different antibody detection methods for diagnosis of COVID-19 is inconclusive.

**Methods:**

Between 16 February and 28 February 2020, 384 confirmed COVID-19 patients and 142 healthy controls were recruited. 24 different serological tests, including 4 enzyme-linked immunosorbent assays (EIAs), 10 chemiluminescent immunoassays (CLIAs), and 10 lateral flow immunoassays (LFIAs), were simultaneously performed.

**Results:**

The sensitivities of anti-SARS-CoV-2 IgG and IgM antibodies with different reagents ranged from 75 to 95.83% and 46.09 to 92.45%, respectively. The specificities of both anti-SARS-CoV-2 IgG and IgM were relatively high and comparable among different reagents, ranged from 88.03 to 100%. The area under the curves (AUCs) of different tests ranged from 0.733 to 0.984, and the AUCs of EIAs or CLIAs were significantly higher than those of LFIAs. The sensitivities of both IgG and IgM gradually increased with increase of onset time. After 3–4 weeks, the sensitivities of anti-SARS-CoV-2 IgG were maintained at a certain level but the sensitivities of IgM were gradually decreased. Six COVID-19 patients who displayed negative anti-SARS-CoV-2 results were associated with the factors such as older age, having underlying diseases, and using immunosuppressant.

**Conclusion:**

Besides the purpose of assessing the impact of the SARS-CoV-2 pandemic in the population, SARS-CoV-2 antibody assays may have an adjunct role in the diagnosis and exclusion of COVID-19, especially by using high-throughput technologies (EIAs or CLIAs).

## Introduction

Coronavirus disease 2019 (COVID-19), the emerging infectious disease caused by a novel severe acute respiratory syndrome coronavirus 2 (SARS-CoV-2), is the greatest threat to public health worldwide in recent 2 years (Phelan et al., 2020; The, 2020; Zhu et al., 2020). Globally, as of February 11, 2022, there have been 404 million confirmed cases of COVID-19, including 5.7 million deaths, reported to the World Health Organization. To this day, COVID-19 pandemic is still the most critical problem in the global health agenda.

The rapid and accurate diagnosis of COVID-19 is the key to control the epidemic of this disease. The diagnosis of COVID-19 is mainly based on epidemiology, clinical symptoms, radiology, and laboratory pathogen detection. The clinical symptoms of COVID-19 include many typical respiratory manifestations (fever, cough, chest pain, or shortness of breath) and other manifestations (fever, muscle ache, fatigue, diarrhea, or headache) (Baj et al., 2020; Brendish et al., 2020; Pan et al., 2020), which are similar to that of influenza (Wang et al., 2014). The asymptomatic COVID-19 patients were also reported previously, increasing the difficulty of diagnosis based solely on clinical features (Rothe et al., 2020; Ralli et al., 2021; Temkin and Healthcare Worker COVID-19 Surveillance Working Group, 2021). The characteristics of radiology of COVID-19 are also unspecific and the diagnosis of which based on radiology has variation among different radiologists (Chung et al., 2020; Kuo et al., 2021). The positive detection of SARS-CoV-2 virus nucleic acid by using real-time reverse transcription polymerase chain reaction (RT-PCR) is the most important diagnostic tool for COVID-19 (Islam and Iqbal, 2020; Pascarella et al., 2020; Zowawi et al., 2021). However, nucleic acid testing has some limitations, such as requiring certified laboratories, experienced technicians and expensive equipment, long turnaround time, and the existence of false negative results (Liu et al., 2020; Sule and Oluwayelu, 2020).

Serological tests are readily available in clinical laboratories in most hospitals and are easier to carry out than molecular tests (Lisboa Bastos et al., 2020; Xiao S.Y. et al., 2020). Besides the purpose of assessing the impact of the SARS-CoV-2 pandemic in the population and evaluating antibody titers from previous exposures to SARS-CoV-2 or from vaccine treatment, serological tests were also recommended to be used for diagnosing or excluding suspected cases in the guideline of diagnosis and treatment for SARS-CoV-2 pneumonia made by Chinese National Health Commission. SARS-CoV-2-specific IgM antibody level peaks at week 3 and then declines, whereas IgG antibodies to spike protein can persist long-term, even beyond 1 year after infection (Gudbjartsson et al., 2020; Hou et al., 2020, 2021; Xiao A.T. et al., 2020). Importantly, although there were many studies focused on the role of SARS-CoV-2-specific antibodies in the diagnosis, prognosis and management of COVID-19 (Lisboa Bastos et al., 2020; Ong et al., 2021), there was rare study to evaluated the performance of different antibody detection methods in clinical practice. Given there are many commercially available anti-SARS-CoV-2 IgM and IgG detection kits developed, it is necessary to verify the accuracy of serological tests in the diagnosis of COVID-19 in clinical practice.

In the current study, we compared the performance of 24 different serological tests, which were classified as enzyme-linked immunosorbent assay (ELISA or EIA), chemiluminescent immunoassay (CLIA), and point-of-care testing (POCT) technology such as lateral flow immunoassay (LFIA), in the diagnosis of COVID-19 patients. We also analyzed the sensitivity of these methods in patients at different time after disease onset. This study is useful for developing the standards for antibody testing and further understanding the appearance and persistence of SARS-CoV-2 antibodies in COVID-19 patients.

## Materials and Methods

### Patients

Between 16 February and 28 February 2020, a total number of 384 COVID-19 patients and 142 healthy controls were continuously recruited from Tongji Hospital (the largest hospital in central region of China), Wuhan, China. The diagnosis of COVID-19 was according to the guideline of diagnosis and treatment for SARS-CoV-2 pneumonia made by Chinese National Health Commission. The confirmed COVID-19 patients were defined as having positive SARS-CoV-2 real-time RT-PCR results in clinical samples, together with typical clinical symptoms (fever, cough, or shortness of breath) and radiological characteristics (unilateral pneumonia, bilateral pneumonia, or ground-glass opacity). The healthy controls were defined as individuals without signs or symptoms of active disease by clinical interview and physical examination, and with negative SARS-CoV-2 real-time RT-PCR results. Five milliliter of venous blood from each participant were collected into a test tube for serum separation, which was then stored at –80°C until use. The serum was thawed and mixed before measurement. The demographic and clinical information, and laboratory results were collected from electronic medical records. This study was approved by the ethical committee of Tongji Hospital, Tongji Medical College, Huazhong University of Science and Technology (TJ-C20200128).

### Severe Acute Respiratory Syndrome Coronavirus 2-Specific Antibody Detection

#### ELISAs

Four ELISA kits were obtained from Livzon [Anti-SARS-CoV-2 ELISA Kit (IgG), Anti-SARS-CoV-2 ELISA Kit (IgM), Livzon Pharmaceutical Group Inc., Zhuhai, China] (Livzon-EIA-IgG, Livzon-EIA-IgM) or WANTAI [Anti-SARS-CoV-2 ELISA Kit (Ab total), Anti-SARS-CoV-2 ELISA Kit (IgM), WANTAI BioPharm Group Inc., Beijing, China] (WANTAI-EIA-Ab total, WANTAI-EIA-IgM) respectively, and performed according to the manufacturers’ instructions. Briefly, –80°C stored serum samples were thawed and mixed before use. After that, the serum samples and reagents were added to 96-well microtiter plates pre-coated with SARS-CoV-2-specific antigens and detected by automated analyzers. Signal to cutoff value (S/CO) ≥ 1 is reactive (positive), and S/CO < 1 is non-reactive (negative).

#### Chemiluminescent Immunoassays

Similarly, –80°C stored serum samples were thawed and mixed before use. Ten anti-SARS-CoV-2 CLIA kits were obtained from InnoDx (InnoDx biotechnology Co., Ltd., Xiamen, China) (InnoDx-CLIA-Ab total, InnoDx-CLIA-IgM), Beier (Beier bioengineering Co., Ltd., Beijing, China) (Beier-CLIA-IgG, Beier-CLIA-IgM), YHLO (YHLO Biotech Co., Ltd., Shenzhen, China) (YHLO-CLIA-IgG, YHLO-CLIA-IgM), Orienter (Orienter Biotechnology Co., Ltd. Chengdu, China) (Orienter-CLIA-IgG, Orienter-CLIA-IgM), and Maccura (Maccura Biotechnology Co., Ltd., Chengdu, China) (Maccura-CLIA-IgG, Maccura-CLIA-IgM), respectively, and performed by automated chemiluminescence analyzers (Caris200, InnoDx; VI-180, Beier; iFlash 3000-C, YHLO; LA2000, Orienter; i 3000, Maccura). For InnoDx, Orienter and Maccura, S/CO ≥ 1 is reactive (positive), and S/CO < 1 is non-reactive (negative). For YHLO, the results ≥ 10 AU/ml is reactive (positive), and the results <10 AU/ml is non-reactive (negative). The cutoff value is 5 RU/ml for Beier.

#### Lateral Flow Immunoassays

Ten POCT anti-SARS-CoV-2 LFIA kits were obtained from Livzon (Livzon-LFIA-IgG, Livzon-LFIA-IgM), WANTAI (WANTAI-LFIA-IgG, WANTAI-LFIA-IgM), Beier (Beier-LFIA-IgG, Beier-LFIA-IgM), HEALGEN (Orient Gene Biotech Co., Ltd., Huzhou, China) (HEALGEN-LFIA-IgG, HEALGEN-LFIA-IgM), and Innovita (Innovita Biological Technology Co., Ltd., Beijing, China) (Innovita-LFIA-IgG, Innovita-LFIA-IgM), respectively, and performed according to the manufacturers’ instructions. Briefly, –80°C stored serum samples were thawed and mixed before use. After that, the serum samples were diluted and added to colloidal gold immunochromatographic strip. The results were finally read by the eyes.

For the antibody detection reagents, YHLO-CLIA-IgG, YHLO-CLIA-IgM, HEALGEN-LFIA-IgG, and HEALGEN-LFIA-IgM have received CE certification in Europe, and InnoDx-CLIA-Ab total, Innovita-LFIA-IgG, and Innovita-LFIA-IgM have been approved by the China National Medical Product Administration.

### Statistical Analysis

Data analyses were performed with SPSS 21.0 (SPSS. Inc.) or GraphPad Prism 6.0.1 (GraphPad). Unless otherwise specified, the data are expressed as mean ± standard deviation (SD). Continuous variables were compared with Mann-Whitney *U*-test. Receiver-operator characteristic (ROC) curve analysis was used to compare the performance of different anti-SARS-CoV-2 assays for diagnosis of COVID-19. The area under the curve (AUC), sensitivity, specificity, positive predictive value (PPV), negative predictive value (NPV), together with 95% confidence interval (CI), were identified. Statistical significance was determined as *p* < 0.05.

## Results

### Participant Characteristics

A total of 384 COVID-19 patients (males, 197; females, 187) were enrolled in this study. The median age was 65 years (range 5–91 years). The median time from onset of symptoms to antibody detection was 21 days (range 3–78 days). A total of 142 healthy individuals (males, 80; females, 62) who tested negative for SARS-CoV-2 were enrolled as control subjects. The median age of healthy controls was 42 years (range 2–90 years).

### The Performance of 24 Severe Acute Respiratory Syndrome Coronavirus 2 Antibody Assays for Coronavirus Disease-2019 Diagnosis

The sensitivity and specificity of these 24 antibody reagents are shown in Table 1. The sensitivities of anti-SARS-CoV-2 IgG or total antibodies with different reagents ranged from 75 to 95.83%. The sensitivities of anti-SARS-CoV-2 IgM ranged from 46.09 to 92.45%. The specificities of both anti-SARS-CoV-2 IgG and IgM with different reagents were relatively high, ranged from 88.03 to 100%. The specificities of IgG and IgM in most reagents were comparable. Our data showed that most commercially available anti-SARS-CoV-2 detection kits, especially the high-throughput technologies (EIAs or CLIAs), have relatively high sensitivity and specificity for COVID-19 diagnosis.

**TABLE 1:** The sensitivity and specificity of 24 SARS-CoV-2 antibody assays.

	Sensitivity (%)	Positive/Total	Specificity (%)	Negative/Total	Positive predictive value (%)	Negative predictive value (%)
Livzon-EIA-IgG	92.19	354/384	96.48	137/142	98.61	82.04
WANTAI-EIA-Ab total	95.83	368/384	97.18	138/142	98.92	89.61
InnoDx-CLIA-Ab total	93.49	359/384	99.30	141/142	99.72	84.94
Beier-CLIA-IgG	75.00	288/384	99.30	141/142	99.65	59.49
YHLO-CLIA-IgG	95.05	365/384	88.03	125/142	95.55	86.81
Orienter-CLIA-IgG	94.27	362/384	97.18	138/142	98.91	86.25
Maccura-CLIA-IgG	92.19	354/384	100	142/142	100	82.56
Livzon-LFIA-IgG	92.71	356/384	99.30	141/142	99.72	83.43
WANTAI-LFIA-IgG	83.33	320/384	95.77	136/142	98.16	68.00
Beier-LFIA-IgG	81.34	231/284*	100	142/142	100	72.82
HEALGEN-LFIA-IgG	91.93	353/384	100	142/142	100	82.08
INNOVITA-LFIA-IgG	92.97	357/384	99.30	141/142	99.73	84.43
Livzon-EIA-IgM	47.14	181/384	99.30	141/142	99.45	40.99
WANTAI-EIA-IgM	85.68	329/384	97.89	139/142	99.10	71.65
InnoDx-CLIA-IgM	89.58	344/384	99.30	141/142	99.71	77.90
Beier-CLIA-IgM	64.32	247/384	97.18	138/142	98.41	50.18
YHLO-CLIA-IgM	84.11	323/384	90.85	129/142	96.13	67.89
Orienter-CLIA-IgM	90.36	347/384	97.89	139/142	99.14	78.98
Maccura-CLIA-IgM	92.45	355/384	100	142/142	100	83.04
Livzon-LFIA-IgM	46.09	177/384	100	142/142	100	40.69
WANTAI-LFIA-IgM	57.81	222/384	99.30	141/142	99.55	46.53
Beier-LFIA-IgM	71.48	203/284*	100	142/142	100	63.68
HEALGEN-LFIA-IgM	69.79	268/384	100	142/142	100	55.04
INNOVITA-LFIA-IgM	67.71	260/384	97.18	138/142	98.48	52.67

*Due to a shortage of reagents or samples, only 284 cases of COVID-19 were detected by Beier-LFIA-IgG/IgM.

**FIGURE 1:**
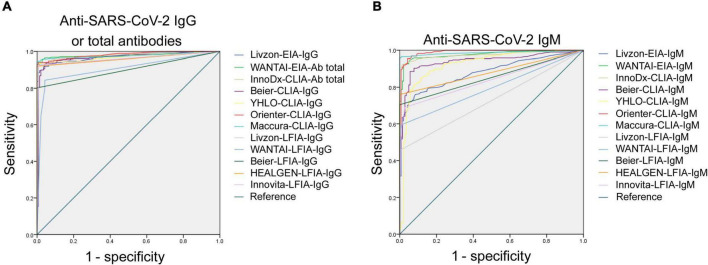
ROC analysis of 24 SARS-CoV-2 antibody assays. **(A)** ROC analysis of anti-SARS-CoV-2 IgG or total antibodies with different reagents. AUCs were 0.973 (95% CI, 0.961–0.985), 0.974 (95% CI, 0.957–0.990), 0.979 (95% CI, 0.968–0.990), 0.969 (95% CI, 0.953–0.986), 0.972 (95% CI, 0.959–0.985), 0.984 (95% CI, 0.976–0.993), 0.980 (95% CI, 0.968–0.991), 0.958 (95% CI, 0.941–0.976), 0.899 (95% CI, 0.871–0.927), 0.901 (95% CI, 0.872–0.930), 0.960 (95% CI, 0.943–0.979), 0.962 (95% CI, 0.945–0.979), and 0.968 (95% CI, 0.954–0.983) for Livzon-EIA-IgG, WANTAI-EIA-Ab total, InnoDx-CLIA-Ab total, Beier-CLIA-IgG, YHLO-CLIA-IgG, Orienter-CLIA-IgG, Maccura-CLIA-IgG, Livzon-LFIA-IgG, WANTAI-LFIA-IgG, Beier-LFIA-IgG, HEALGEN-LFIA-IgG, and Innovita-LFIA-IgG, respectively. **(B)** ROC analysis of anti-SARS-CoV-2 IgM with different reagents. AUCs were 0.865 (95% CI, 0.835–0.896), 0.976 (95% CI, 0.964–0.988), 0.974 (95% CI, 0.962–0.987), 0.946 (95% CI, 0.926–0.966), 0.927 (95% CI, 0.900–0.955), 0.984 (95% CI, 0.973–0.995), 0.979 (95% CI, 0.966–0.991), 0.733 (95% CI, 0.692–0.774), 0.787 (95% CI, 0.750–0.824), 0.852 (95% CI, 0.817–0.887), 0.849 (95% CI, 0.818–0.880), and 0.831 (95% CI, 0.797–0.864) for Livzon-EIA-IgM, WANTAI-EIA-IgM, InnoDx-CLIA-IgM, Beier-CLIA-IgM, YHLO-CLIA-IgM, Orienter-CLIA-IgM, Maccura-CLIA-IgM, Livzon-LFIA-IgM, WANTAI-LFIA-IgM, Beier-LFIA-IgM, HEALGEN-LFIA-IgM, and Innovita-LFIA-IgM, respectively. CI, confidence interval.

**FIGURE 2:**
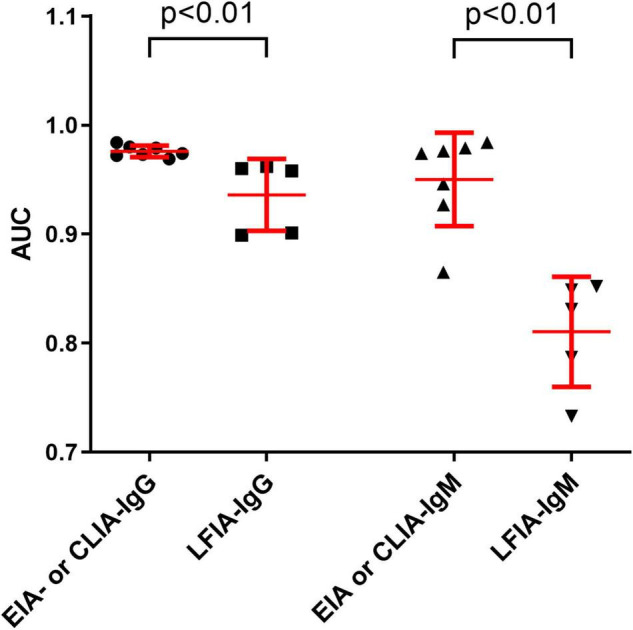
Comparison of the AUCs between EIA- or CLIA-IgG and LFIA-IgG, and between EIA- or CLIA-IgM and LFIA-IgM. Data were expressed as mean and standard deviation. AUC, area under the curve.

ROC analysis for each assay was determined. As shown in Figure 1, the AUCs of these 24 assays ranged from 0.733 to 0.984. The AUCs of EIAs or CLIAs, no matter in IgG or IgM, were significantly higher than those of LFIAs (Figure 2). The AUCs of these 24 assays ranged in a descending order from: Orienter-CLIA-IgG > Maccura-CLIA-IgG > InnoDx-CLIA-Ab total > WANTAI-EIA-Ab total > Livzon-EIA-IgG > YHLO-CLIA-IgG > Beier-CLIA-IgG > Innovita-LFIA-IgG > HEALGEN-LFIA-IgG > Livzon-LFIA-IgG > Beier-LFIA-IgG > WANTAI-LFIA-IgG for IgG or total antibody detection; and Orienter-CLIA-IgM > Maccura-CLIA-IgM > WANTAI-EIA-IgM > InnoDx-CLIA-IgM >Beier-CLIA-IgM > YHLO-CLIA-IgM > Livzon-EIA-IgM > Beier-LFIA-IgM > HEALGEN-LFIA-IgM > Innovita-LFIA-IgM > WANTAI-LFIA-IgM > Livzon-LFIA-IgM for IgM detection.

**FIGURE 3:**
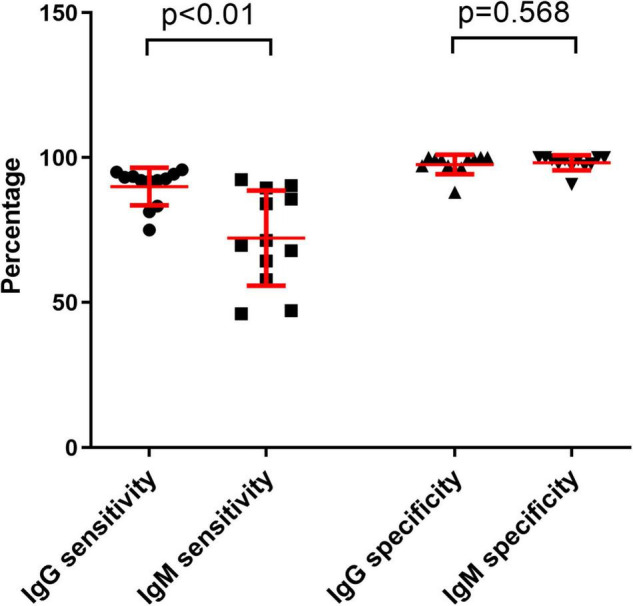
Comparing the differences between IgG sensitivity and IgM sensitivity or between IgG specificity and IgM specificity with different reagents. Data were expressed as mean and standard deviation.

**FIGURE 4:**
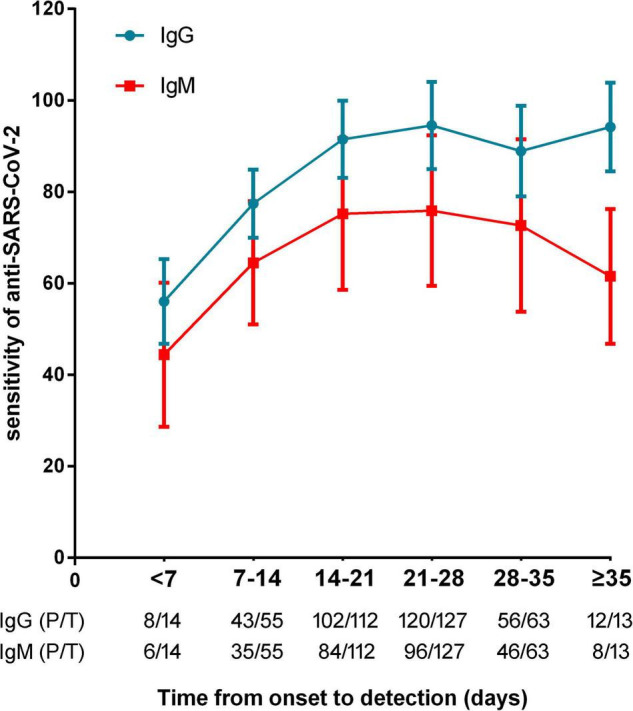
The sensitivities of anti-SARS-CoV-2 antibodies in patients with different timepoints (calculated from onset of symptoms to antibody detection). P/T, positive number/total number.

The sensitivities of anti-SARS-CoV-2 IgM were significantly lower than those of anti-SARS-CoV-2 IgG or total antibodies (*p* < 0.01), but the specificities between anti-SARS-CoV-2 IgG and IgM had no difference (*p* = 0.568) (Figure 3). The sensitivities of both IgG and IgM were gradually increased with increase of onset time, and reached the peak after about 3–4 weeks. After that, the sensitivities of anti-SARS-CoV-2 IgG were maintained at a certain level but the sensitivities of anti-SARS-CoV-2 IgM were gradually decreased (Figure 4).

### False-Negative Results of Severe Acute Respiratory Syndrome Coronavirus 2 Antibody Assays

There were 6 COVID-19 patients who had negative anti-SARS-CoV-2 results by any detection kits. We observed that days from onset to antibody detection were between 3 and 12 days in these patients. Furthermore, five of six patients had underlying diseases such as hypertension, diabetes, and coronary heart disease. One patient with systemic lupus erythematosus was undergoing immunosuppressant treatment (rituximab). The demographic and clinical characteristics of these patients are shown in Table 2. These data suggested that the false negative results of anti-SARS-CoV-2 results may be caused by the variability in the time from onset of illness to detection or the immunosuppression status in COVID-19 patients.

**TABLE 2:** The demographic and clinical characteristics of 6 COVID-19 patients with negative anti-SARS-CoV-2 results in all 24 assays.

No.	Sex	Age (years)	Time from onset to detection (days)	Severity	Underlying disease
19	Female	75	12	Severe	Hypertension, coronary heart disease
162	Male	34	5	Mild	None
191	Female	22	7	Extremely severe	Systemic lupus erythematosus, using immunosuppressant (rituximab)
208	Female	27	3	Mild	Hyperthyreosis, pregnancy
219	Female	66	7	Severe	Hypertension, diabetes, coronary heart disease, endometrial cancer (after surgery)
236	Male	57	3	Severe	Hypertension, diabetes, lung cancer (after surgery)

## Discussion

The early diagnosis and isolation of COVID-19 patients are the key to control the outbreak of the disease. Given the false-negative results of SARS-CoV-2 RT-PCR is common in clinical samples, especially in patients with increased time since symptom onset or with oropharyngeal samples rather than nasopharyngeal samples, it is unsuitable for use of the method to exclude COVID-19 (Arevalo-Rodriguez et al., 2020; Wikramaratna et al., 2020). With the outbreak of COVID-19, many SARS-CoV-2 antibody detection methods based on different methodologies such as ELISA, CLIA and LFIA have been developed. The current view emphasizes that SARS-CoV-2 antibodies serve as an complement to RT-PCR in the diagnosis of acute infection (Sidiq et al., 2020). However, the performance of these antibody detection methods for the diagnosis of COVID-19 patients is inconclusive. In this study, we compared the performance of almost all current commercially available assays for anti-SARS-CoV-2 detection in China. Our data showed that the performance of high-throughput technologies including EIAs and CLIAs was superior to POCT. Moreover, most EIAs and CLIAs had high sensitivity and specificity and comparable diagnostic accuracy, which confirmed that SARS-CoV-2 antibody detection may have an adjunct role in the diagnosis of COVID-19.

Regarding using serological tests for COVID-19 diagnosis, there were two main aspects that should be considered. First, the test should have enough sensitivity and specificity to facilitate COVID-19 diagnosis. Second, technical efficiency and bio-safety also counted. Our data showed that most anti-SARS-CoV-2 IgG tests and many anti-SARS-CoV-2 IgM tests such as Orienter-CLIA-IgM and Maccura-CLIA-IgM, achieved over 90% sensitivity in the diagnosis of COVID-19, which was in accordance with previous study (Li et al., 2020). Thus, SARS-CoV-2 antibody detection was of important value in early diagnosis of COVID-19, especially in patients suspected as SARS-CoV-2 infection but with negative RT-PCR results. On the other hand, both anti-SARS-CoV-2 IgG and IgM detection had high specificity (mostly higher than 95%) for diagnosis of COVID-19. Hereafter, these data suggest that SARS-CoV-2 antibody detection plays an important role in the diagnosis and exclusion of COVID-19 patients.

Except for Beier, the sensitivities of all other anti-SARS-CoV-2 IgG detection methods were relatively high. Nevertheless, the sensitivities of anti-SARS-CoV-2 IgM detection methods varied greatly, and the sensitivities of WANTAI-EIA-IgM, InnoDx-CLIA-IgM, YHLO-CLIA-IgM, Orienter-CLIA-IgM and Maccura-CLIA-IgM were higher than those of others. Overall, the sensitivities of anti-SARS-CoV-2 IgG in different methods were higher than IgM, but the specificity of both anti-SARS-CoV-2 IgG and IgM had no difference among these methods. Generally, antigen-specific IgM can be early detected after pathogen infection and then rapidly decreases in several weeks. In contrast, IgG usually appears later but maintains at a certain level for a long time. Consistent with this notion, it was reported that IgM could be detected in peripheral blood of COVID-19 patients after 3–7 days and that IgG could be detected after 7–8 days (Zhou et al., 2020). It is worthy to note that, the median time from onset to antibody detection was 21 days in the present study, and we speculated that our enrolled patients were in the middle stage of infection or recovery period. This could be used to explain the low sensitivity of anti-SARS-CoV-2 IgM in these patients. Our findings indicated that the COVID-19 patients with decreased level of IgM but with maintained level of IgG may be in the status of recovery. These data suggest that SARS-CoV-2 antibody detection could also play an important role in the treatment monitoring and prognosis of COVID-19.

Due to the highly contagious nature of the disease, even asymptomatic carriers could spread SARS-CoV-2 virus, which made the control of COVID-19 outbreak more difficult (Rothe et al., 2020; Zou et al., 2020). Given that the sensitivities of SARS-CoV-2 antibody assays were high, these assays had great value in screening asymptomatic SARS-CoV-2 carriers. However, we still observed 6 COVID-19 patients with false-negative results of all antibody detection methods (Table 2). The reasons could be as follows. First, low concentration of antibodies could lead to false negative results. As shown in Table 2, the days from onset to antibody detection of five patients were within 7 days, while IgM and IgG levels may be below the detection limit during this period. Second, the heterogeneity of immune response to SARS-CoV-2 in different individuals may cause delayed antibody production in some individuals. Third, the patients with underlying conditions may be one of the important reasons contributing to false-negative results of antibody detection. As shown in Table 2, one patient (No. 19) was a seventy-five-year-old female who might have impaired immunity because of older age (Chandra, 2002). Another three patients (No. 191, No. 219, No. 236) had immunocompromised conditions such as diabetes, lung cancer, and undergoing immunosuppressant treatment, which could affect the producing of antibodies in these patients. In addition, previous study has shown that the possibility of false-negative results of SARS-CoV-2 antibody assays should be considered if the sample was pre-inactivated by heating, which suggests that heat inactivation prior to immunoanalysis is not recommended (Hu et al., 2020). On the other hand, the false-positive results of SARS-CoV-2 antibody assays maybe due to cross-reactivity with anti-HBV, anti-influenza, and rheumatoid factor (Tre-Hardy et al., 2020).

Some limitations of the study should be mentioned. First, since Tongji Hospital was one of designated hospitals for transfer of patients with COVID-19 from other hospitals, the enrolled patients in this study had a relatively prolonged time from onset of symptoms to admission. This is the reason why the median time from onset to antibody detection was 21 days, which could affect the results of antibody detection. Second, we did not continuously monitor the producing of antibodies in the same patients, and further study is needed.

Taken together, the present study confirms that SARS-CoV-2 antibody assays have good performance in the diagnosis and exclusion of COVID-19 patients, especially by using high-throughput technologies (EIAs or CLIAs), which suggests that antibody detection of SARS-CoV-2 may play an important role in the control of COVID-19.

## Data Availability Statement

The original contributions presented in this study are included in the article/supplementary material, further inquiries can be directed to the corresponding authors.

## Ethics Statement

This study was approved by the ethical committee of Tongji Hospital, Tongji Medical College, Huazhong University of Science and Technology (TJ-C20200128). Written informed consent for participation was not required for this study in accordance with the national legislation and the institutional requirements.

## Author Contributions

SW and FW wrote the first draft of the manuscript and involved in data curation and project administration. HL managed reagents. HH and TW researched the literature and conceived the study. WW and MZ collected the specimen. SW and BY involved in experiment operation. MH, ZS, and FW involved in protocol development, gaining ethical approval, patient recruitment, and data analysis. All authors reviewed and edited the manuscript and approved the final version of the manuscript.

## Conflict of Interest

The authors declare that the research was conducted in the absence of any commercial or financial relationships that could be construed as a potential conflict of interest.

## Publisher’s Note

All claims expressed in this article are solely those of the authors and do not necessarily represent those of their affiliated organizations, or those of the publisher, the editors and the reviewers. Any product that may be evaluated in this article, or claim that may be made by its manufacturer, is not guaranteed or endorsed by the publisher.
